# Uterine Displacement

**Published:** 1872-12

**Authors:** L. E. Wells

**Affiliations:** Phenix, R. I.


### UTERINE DISPLACEMENT.

BY L. E. WELLS, M.D.

On the 1st instant I was consulted by Mrs. J., aged 37, and the mother of three children. She informed me that about the 12th of last December, she suffered from a miscarriage, having advanced about three months on her fourth gestation. At that time, she became very much reduced by flowing. Since then there has been constant tenderness, and at times severe pain in all the region of the womb, but more especially in the vicinity of the left ovary. There has also been, for the most of the time, a discharge of whites. After hearing her history of the case, I made a vaginal examination, which revealed to me the fact of the existence of acute tenderness throughout all that portion of the sexual organs which is susceptible to an examination by the touch. But what was of the chief interest to me was this: The uterus had been raised from its ordinary position and rested directly upon the summit of the bladder, and this organ was depressed so that the cervix uteri could be felt low down, near the base of the former, and between the womb and finger were the walls of the bladder.

This is to me a singular case, and in all my experience as a gynecologist, I have never met with anything like it.

Therefore, two questions arise in my mind pertinent to this case. First, will it be expedient to try to bring back to its natural position, the displaced womb? Second, if it be expedient to do this, what will be the proper course to pursue?—*Boston Med. & Surg. Journal.*

Phenix, R. I., August 17, 1872.

